# Urinary and serum benzodiazepine determinations and its correlation with the sedation wakening in critical care patients

**DOI:** 10.1186/2197-425X-3-S1-A330

**Published:** 2015-10-01

**Authors:** S Rosich Andreu, G Moreno Muñoz, J Marin Corral, E Machado, R Sanchez, À Vilanova, M Olono, MA Bodí, AH Rodríguez, A Sandiumenge Camps

**Affiliations:** Critical Care Department, Joan XXIII, University Hospital, Tarragona, Spain; Joan XXIII, University Hospital, Laboratory, Tarragona Spain; Preventive Department, Joan XXIII, University Hospital, Tarragona, Spain; CIBERES (CIBER Enfermedades Respiratorias), Bunyola, Spain; IISPV-URV, Tarragona, Spain; Vall d'Hebron University Hospital-VHIR, Barcelona, Spain

## Introduction

The accumulation of benzodiazepines (BZD) or their metabolites in critically ill patients may prolong wakening times after its discontinuation increasing their morbidity and leading to the overuse of complementary testing to assess neurological function. Route urine/serum determination of BZD are used in some centers to determine BZD clearance, however the correlation of BZD urine/blood concentration and its clinical action has not been determined in critically ill patients.

## Objectives

We aimed to investigate the correlation between the presence of BZD in urine and blood (midazolam-MDZ) with the time of wakening after discontinuation of prolonged intravenous (iv) infusion of MDZ in critical care patients. We also aimed to determine the impact of renal failure (ARF) in the efficacy of those test.

## Methods

Prospective, observational study in a 30-bed adult medical-surgical intensive care unit (ICU) from Oct 2014 to Apr 2015. All patients admitted with GCS>8 who were sedated with iv MDZ infusion >72 hours were followed from sedation discontinuation until complete wakening. Patients with chronic use of BZD previous to ICU admission or treated with oral BZD after MDZ iv discontinuation were excluded. Daily urine qualitative (One step rapid test, Healgen®') and blood quantitative (Advia Chemistry, Siemens®) BZD determinations were taken until the first negative urine BZD result. Renal function was determined using RIFLE scale. Time to wake up was defined as the number of hours from BZD discontinuation to the ability to obey simple commands.

## Results

A total of 32 patients were included, 8 of whom were excluded due to chronic use of BZD (2[25%]) or treatment with oral BZD after MDZ iv discontinuation (6[75%]). The remaining 24 patients (male 70%, 63.3(SD 12) years old, APACHE II 23(SD 8), SAPS 3 40(SD 22) were sedated with 2382.32mg (SD 3402.20) of MDZ during 7(SD 6) days.A strong correlation between MDZ serum levels and urine BZD positivity (p < 0.0001,R² 0.89) was found. We also found an statistically significant correlation between the number of days of positive BZD urinary determinations and the time to wake up (p = 0.002), but with a low clinical relevance (R² 0.392). Discrimination power (ROC) of MDZ serum levels to predict the wakening showed an AUC of 0.71 (0.61-0.80, p = 0.001).Patients with ARF (14 [58%]) had more days of positive BZD urinary determinations compared with the group without ARF (10[41%])(10.5[SD 7.8]vs.4.6[SD 4.9],p = 0.032), despite receiving lower total doses of MDZ (2245.72mg [SD 2589.88] vs. 2573.57mg [SD 4452.21],p = 0.838) respectively.

## Conclusions

Qualitative BZD urine determination is not a useful tool to predict wake up times in critically ill patients receiving prolonged MDZ infusions. The determination of serum levels of midazolam can readily predict the wakening time in more than 70% of ICU patients who had received long-term MDZ sedation.

